# Rapid *in vivo *analysis of synthetic promoters for plant pathogen phytosensing

**DOI:** 10.1186/1472-6750-11-108

**Published:** 2011-11-17

**Authors:** Wusheng Liu, Mitra Mazarei, Mary R Rudis, Michael H Fethe, C Neal Stewart

**Affiliations:** 1 Department of Plant Sciences, The University of Tennessee, 252 Ellington Plant Sciences, 2431 Joe Johnson Dr., Knoxville, TN 37996, USA

## Abstract

**Background:**

We aimed to engineer transgenic plants for the purpose of early detection of plant pathogen infection, which was accomplished by employing synthetic pathogen inducible promoters fused to reporter genes for altered phenotypes in response to the pathogen infection. Toward this end, a number of synthetic promoters consisting of inducible regulatory elements fused to a red fluorescent protein (RFP) reporter were constructed for use in phytosensing.

**Results:**

For rapid analysis, an *Agrobacterium*-mediated transient expression assay was evaluated, then utilized to assess the inducibility of each synthetic promoter construct *in vivo*. Tobacco (*Nicotiana tabacum *cv. Xanthi) leaves were infiltrated with *Agrobacterium *harboring the individual synthetic promoter-reporter constructs. The infiltrated tobacco leaves were re-infiltrated with biotic (bacterial pathogens) or abiotic (plant defense signal molecules salicylic acid, ethylene and methyl jasmonate) agents 24 and 48 hours after initial agroinfiltration, followed by RFP measurements at relevant time points after treatment. These analyses indicated that the synthetic promoter constructs were capable of conferring the inducibility of the RFP reporter in response to appropriate phytohormones and bacterial pathogens, accordingly.

**Conclusions:**

These observations demonstrate that the *Agrobacterium*-mediated transient expression is an efficient method for *in vivo *assays of promoter constructs in less than one week. Our results provide the opportunity to gain further insights into the versatility of the expression system as a potential tool for high-throughput *in planta *expression screening prior to generating stably transgenic plants for pathogen phytosensing. This system could also be utilized for temporary phytosensing; e.g., not requiring stably transgenic plants.

## Background

Transgenic techniques have become a powerful tool to address important biological and agricultural challenges, and there is great potential for the production of designer plants using modern biotechnology tools [1,2]. Many of these applications are addressed by the use of transgenic techniques, including the introduction of homologous or heterologous genes in plants with modified functions and altered expression patterns.

The overall goal of our project is to design plants for the purpose of early detection of plant pathogen infection, which we propose would be attainable employing pathogen inducible promoters fused to reporter genes for altered phenotypes in response to the pathogen infection. The use of "tuned" inducible promoters is a key design feature when constructing transgenic plants as "phytosensors." Inducible plant defense is controlled by signal transduction pathways, inducible promoters and *cis*-acting regulatory elements (REs) corresponding to key proteins involved in defense, and pathogen-specific responses. These *cis*-acting regulatory elements are conserved among plant species, enabling them to be used to construct synthetic inducible promoters in heterologous expression systems [3-5]. Upon detection of a pathogen, we expect a gain-of-function response in the form of expression of the visible marker gene.

Our initial study demonstrated the possible utilization of these *cis*-acting regulatory elements in building phytosensors [6]. This prompted us to construct a number of synthetic promoters consisting of selected *cis*-acting regulatory elements fused to a red fluorescent protein *pporRFP *reporter gene (from the hard coral *Porites porites*; [7]) for use in phytosensing. However, examining a suite of synthetic promoter constructs for their suitability and potential applications in phytosensing involves the generation of many stably transgenic plants harboring many different constructs. Although there is no substitute for stable plant transformation for complete transgenic construct characterization, current procedures are time-consuming, laborious, and not suited for high-throughput assays. Alternatively, transient expression through agroinfiltration is a simple and useful method and has been demonstrated to be effective in many plant species [8-19]. The use of transient gene expression assays offer an opportunity to study a large number of transgene constructs rapidly, which particularly would be advantageous for evaluating the transcriptional activity of different promoters and the interaction between transcription factors and *cis*-acting regulatory sequences presented in plant promoters [3,10,12,17,20,21].

In the present study, we have evaluated an *Agrobacterium*-mediated transient expression assay to assess the inducibility of a number of synthetic promoter constructs *in vivo*. Our results demonstrate that this transient expression is a robust and efficient method for *in vivo *assays of promoter constructs in response to biotic and abiotic agents. The use of synthetic promoters combined with the agroinfiltration assay provides a robust screening method to rapidly evaluate plant-pathogen interactions prior to stable plant transformation.

## Results and Discussion

**Figure 1:**
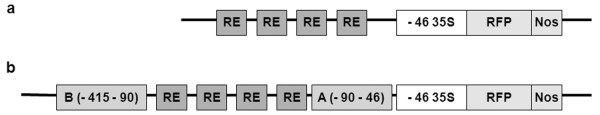
**Scheme of synthetic promoter- *pporRFP *fusion constructs**. (a) Scheme of synthetic promoter construct as tetramers of certain regulatory elements (4 × RE) were placed upstream of CaMV35S minimal promoter (min 35S containing the TATA box). (b) Scheme of enhanced synthetic promoter construct of 4 × RE were placed between B (-415 -90) and A1 (-90 -46) domains of CaMV35S promoter.

**Figure 2:**
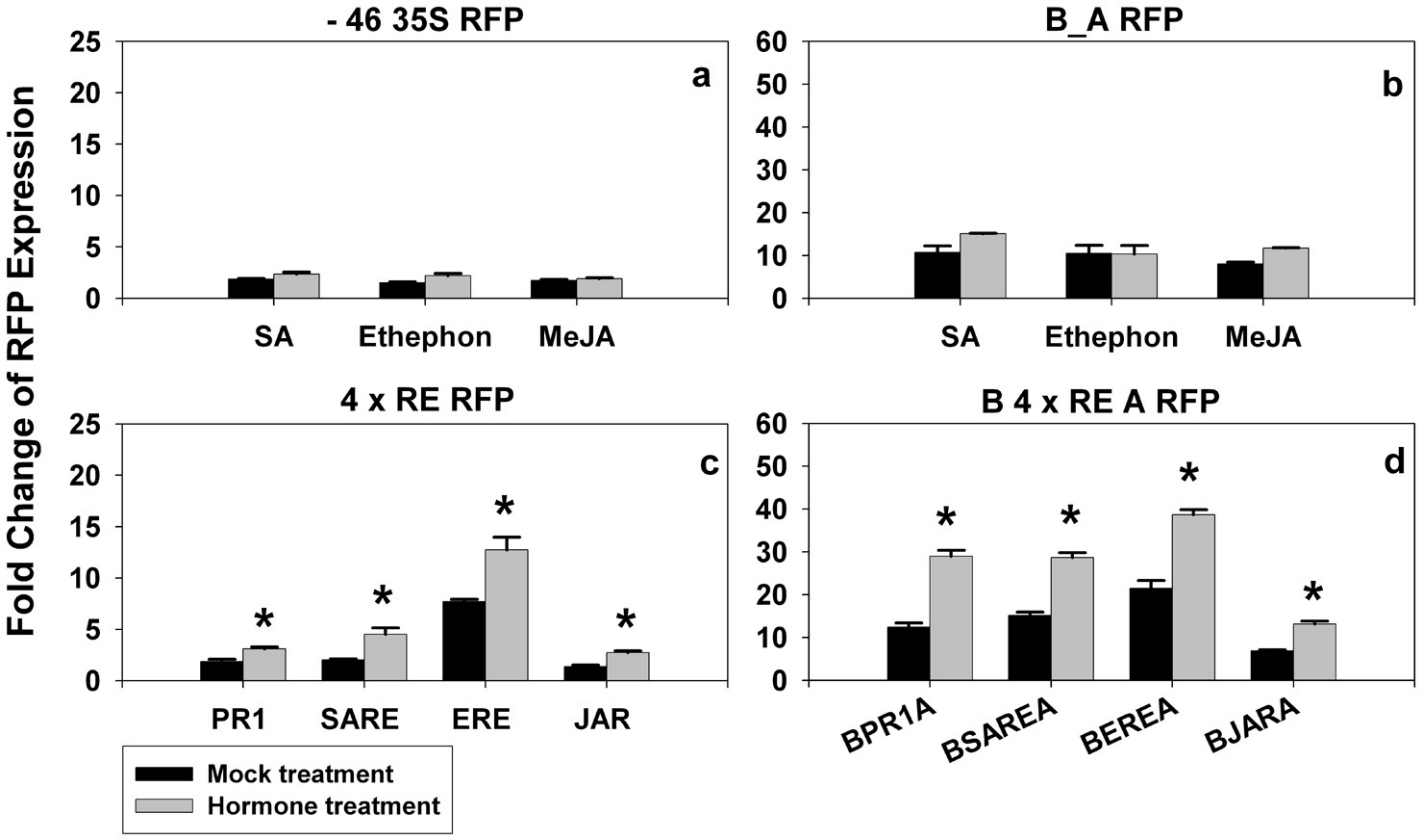
**Fold changes of expression of *pporRFP *reporter following phytohormone treatments**. Forty-eight hours after initial agroinfiltration of individual synthetic promoter constructs, the tobacco leaves were re-infiltrated with mock, salicylic acid (SA) for PR1 and SARE, ethephon (an ethylene releasing chemical) for ERE, and methyl jasmonate (MeJA) for JAR regulatory element containing constructs. Expression of *pporRFP *reporter was quantified using SPEX Fluorolog at time points 0 h (before treatment) and 72 h following treatments. Each bar represents the mean value of *pporRFP *expression obtained from three independent biological experiments with the standard errors of the mean noted. Significant *pporRFP *expression changes (indicated by asterisks) were determined statistically by use of a paired *t *test (*p *< 0.05).

**Figure 3:**
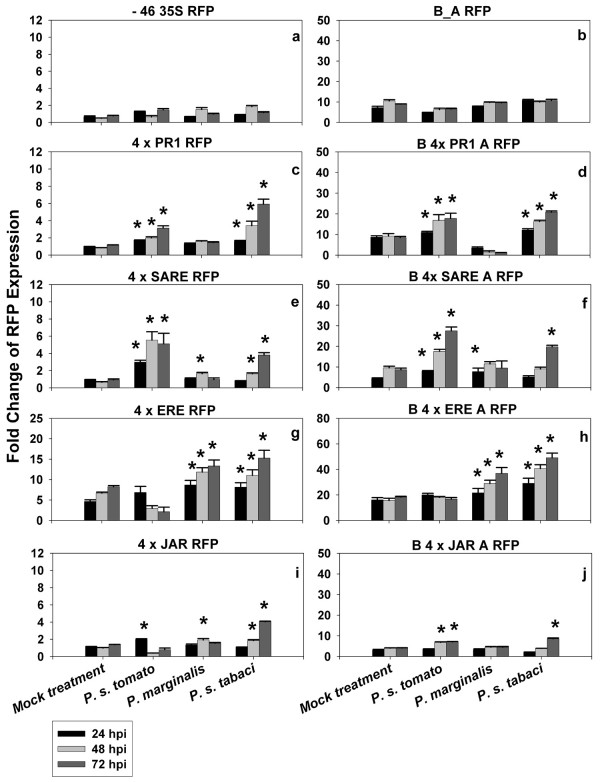
**Fold changes of expression of *pporRFP *reporter following phytopathogenic bacterial treatments**. Twenty-four hours after initial agroinfiltration of individual synthetic promoter constructs, tobacco leaves were re-infiltrated with mock, *P. syringae *pv. *tomato, P. marginalis*, or *P. syringae *pv. *tabaci*. Expression of *pporRFP *reporter was quantified using a SPEX Fluorolog at time points 0, 24, 48, and 72 h post inoculation (hpi). Each bar represents the mean value of *pporRFP *expression obtained from three independent biological experiments with the standard errors of the mean noted. Significant *pporRFP *expression changes (indicated by asterisks) were determined statistically by use of a paired *t-*test (*p *< 0.05).

We constructed a series of synthetic promoter constructs with inducible regulatory elements responding to plant signal defense molecules salicylic acid (SA), ethylene (ET), and jasmonic acid (JA) fused to the *pporRFP *reporter gene, with or without enhancer motifs. Based on our previous results [6], we selected the various *cis*-regulatory elements among the various types of hormone-responsive promoters: SA regulatory elements, PR1 and SARE; JA regulatory elements, JAR; and ET regulatory elements, ERE. Tetramers of the selected regulatory elements were used in each synthetic promoter construct to confer higher inducibility of the regulatory elements [3,6,22]. For increasing basal expression level of the *pporRFP *reporter, one version of the CaMV 35S enhancer was used where the regulatory element tetramer was placed between B (- 415 to-90) and A1 (- 90 to-46) regions of 35S promoter [6] (Figure 1). This version of the enhancer would result in increased basal expression while the induction rate of the synthetic regulatory elements remains nearly the same [23]. To determine the level of background expression associated with the synthetic promoters, the negative controls (empty vectors -46 35S RFP and B_A RFP) were agroinfiltrated into tobacco leaves, followed by treatment with the phytohormones SA, ET and JA or bacterial pathogens *P. syringae *pv. *tomato, P. marginalis *and *P. syringae *pv. *tabaci*. Expression of the *pporRFP *reporter was quantified for both empty vectors at time points 0 (before treatment) and 72 h after phytohormone treatments, or at time points 0, 24, 48, 72 h after bacterial pathogen treatments. As shown in Figure 2a, b and 3a, b, the treatment with either phytohormones or bacterial pathogens caused similar fold changes of *pporRFP *expression in empty vectors as their corresponding mock control treatments and no inducibility of the *pporRFP *expression was observed in either empty vector at any time points compared to their mock treatments.

**Figure 4:**
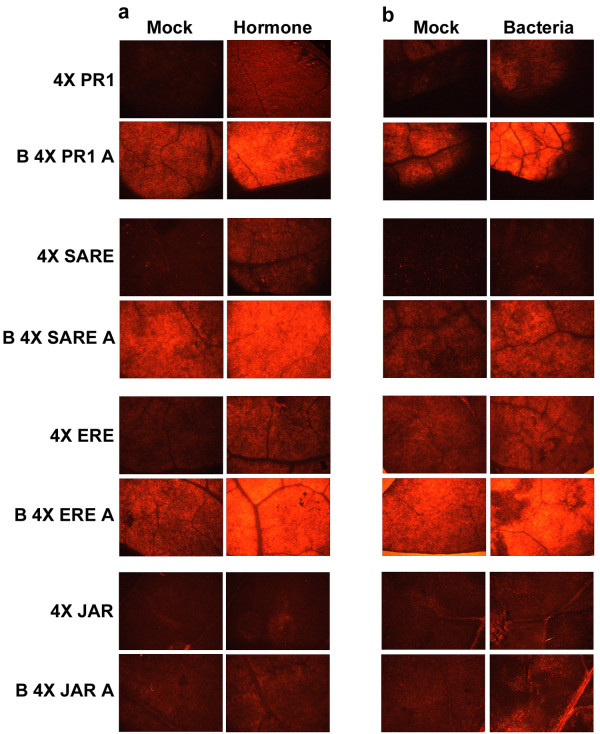
**Visual images (representative) of expression of *pporRFP *reporter following phytohormone or phytopathogenic bacterial treatments**. After initial agroinfiltration of individual synthetic promoter construct, the tobacco leaves were re-infiltrated with (a) salicylic acid for PR1 and SARE, ethephon (an ethylene releasing chemical) for ERE, and methyl jasmonate for JAR regulatory element containing constructs or with (b) *P. syringae *pv. *tabaci*. Expression of *pporRFP *reporter was visualized 72 h following the treatment using epifluorescence microscope Olympus SZX12, and the images were captured using imaging software QCapture 2.56. The exposure time was 1 minute.

Subsequently, the inducibility of the synthetic promoters containing the regulatory elements was examined by phytohormone treatments after initial agroinfiltration of the tobacco leaves. Expression of *pporRFP *reporter in each synthetic promoter construct was measured at time points 0 and 72 h after treatments to be consistent with the time course analysis of bacterial pathogen treatments. In all the experiments, the synthetic promoter constructs showed an increase in *pporRFP *expression at 72 h following their corresponding phytohormone treatment (Figure 2c, d and 4a). The average fold changes of expression of the synthetic promoter constructs caused by their corresponding hormone treatments was 2.3 times higher compared to their respective mock treatments, which were statistically significant at *p *< 0.05. Among the synthetic promoter constructs, 4 × ERE regulatory element conferred the highest induction level while 4 × JAR exhibited the lowest induction level (Figure 2c, d). Furthermore, all the synthetic promoter constructs, regardless of inclusion of B_A enhancers, demonstrated a steady induction level following their corresponding hormone treatments (Figure 2c, d). This observation was consistent with findings by Raventos *et al*. [23] that the presence of the B_A enhancer increased the basal level of the reporter expression but does not change the induction rate of the regulatory element. Our results indicate that these regulatory elements are specifically inducible by their corresponding hormone treatments using this *in vivo *transient agroinfiltration assay.

**Figure 5:**
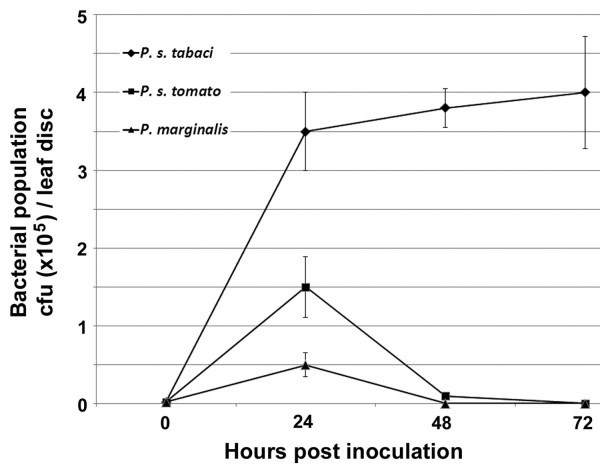
**Growth of bacterial pathogens in tobacco leaves**. Tobacco leaves were infiltrated with *P. syringae *pv. *tabaci, P. syringae *pv. *tomato*, or *P. marginalis*, as described in "Methods." Bacterial population size was determined in leaf discs from inoculated leaves. Data points represent mean colony-forming unit (cfu) per leaf disc of three independent experiments with the standard errors of the mean noted.

Subsequent experiments involving pathogen assays were conducted to analyze both the sensitivity and inducibility of the synthetic promoters against a range of different bacterial pathogen infections. To test whether these synthetic inducible promoters were able to reflect the differences in the expression of the *pporRFP *reporter in a compatible (susceptible) or incompatible (resistance) host-pathogen interaction, we included three phytopathogenic bacteria in our experiments: *P. syringae *pv. *tomato, P. marginalis *and *P. syringae *pv. *tabaci*. We first conducted time-course analysis of tobacco reaction to the bacterial pathogens for resistant and susceptible disease tests accordingly [24-27] in which a resistance hypersensitive response (HR) was evident within 24 hours post inoculation (hpi) and a susceptible normal-sensitive response was evident within 72 hpi. Consistently, our analyses showed that inoculation of tobacco leaves with *P. syringae *pv. *tomato *led to HR associated with the onset of necrosis and dehydration of the tissue (resistance reaction) within 24 hpi, along with a slight and transient increase in bacterial growth that was followed by a dramatic reduction in bacterial population size (Figure 5). Inoculation with *P. syringae *pv. *tabaci *led to a normal-sensitive symptom of "wildfire" disease associated with initial chlorosis within 72 hpi, then subsequently water soaked and necrotic symptom of the tissue (susceptible reaction), along with increase in bacterial growth over the period of time (Figure 5). The non-host resistance of tobacco to *P. marginali*s did not lead to HR reaction and no symptom development was evident following this bacterium inoculation. This result was predicted, since *P. marginali*s is a "soft-rot" disease causing pathogen which does not develop HR reaction in tobacco leaves [28]. Nevertheless, we observed a slight increase, followed by a dramatic reduction, in bacterial population size over the time period after *P. marginali*s infection was evident (Figure 5).

In order to examine the inducubility of the synthetic promoter constructs in response to pathogen infection, tobacco leaves were inoculated with the bacterial pathogens 24 h after initial agroinfiltation of each synthetic promoter construct and a time-course analysis of *pporRFP *expression was conducted. As shown in Figure 3, the synthetic promoter constructs, with or without B_A enhancer, exhibited inducibility in response to *P. syringae *pv. *tomato *within 24-48 hpi. This result, in fact, reflects the rapid HR reaction caused by the bacterial pathogen during the incompatible interaction with tobacco. Yet, at time point 24 hpi, *P. syringae *pv. *tomato *showed a higher inducibility of the *pporRFP *expression in synthetic promoter constructs containing PR1 and SARE regulatory elements. We believe this result was most likely observed because of the fact that the signal transduction of HR reaction is primarily through the SA-dependent pathway. Accordingly, among the regulatory elements, ERE conferred the least inducibility of *pporRFP *expression in response to *P. syringae *pv. *tomato*, implying less association of ethylene signaling with HR reaction. Time course analysis of *pporRFP *expression in response to *P. syringae *pv. *tabaci *indicated a gradual increase of RFP expression over time following bacterial inoculation (Figure 3, 4b). These results reflect the fact that the normal-sensitive "wildfire" disease symptom, caused by the bacterial pathogen during the compatible interaction with tobacco develops within 72 hpi. In response to *P. marginalis *infection, the four regulatory elements individually gave rise to a low level of induction of the *pporRFP *expression at all the time points (Figure 3). The only exception was observed for the ERE regulatory element, which conferred relatively higher induction of the *pporRFP *expression compared to other regulatory elements. Furthermore, depending on the synthetic promoter constructs used, the increase in *pporRFP *expression over time caused by different bacterial pathogens was also variable. For example, as shown in Figure 3, while PR1 and SARE regulatory elements conferred rapid induciblity of *pporRFP *expression at time point of 24 h after *P. syringae *pv. *tomato *infection, ERE showed a low level of inducibility after *P. syringae *pv. *tomato *infection but relatively higher level of induciblity over time after *P. syringae *pv. *tabaci *and *P. marginalis *infections.

Our experimental system hinges on inducible regulation of *cis*-acting elements upon bacterial pathogen exposure for early pathogen detection using *Agrobacterium*-mediated transient assay. Agroinfiltration had been used to study the functional activity of promoters and/or genes during bacterial pathogen, virus, abiotic or environmental stresses [29,30]. In our experiments, the *Agrobacterium*-mediated transient expression was used together with gain-of-function experiments of inducible regulatory elements for its suitability, inducibility and potential applications in early bacterial pathogen phytosensing. We have demonstrated that agroinfiltration of tobacco leaves with synthetic promoter constructs has the potential to validate the inducibility of the regulatory elements in response to the corresponding phytohormone treatment. Moreover, it is capable of examining the responsiveness of the *pporRFP *reporter to bacterial pathogens in both compatible and incompatible interactions. These observations indicate that the *Agrobacterium*-mediated transient expression can be used as a rapid screening tool for *in vivo *analysis of promoter constructs for pathogen phytosensing before conducting stable plant transformation experiments, thus narrowing the most appropriate and effective constructs to use for stable phytosensing experiments. We do not believe it can make absolute predictions for the results of stable transformation; stable transformation involves integration into the plant genome and thus every new transformation event could confer a variety of different synthetic promoter response activities. In addition, this system could also be utilized for temporary phytosensing without the need for deploying stable transformants, since levels of transient expression may be much higher than that of stable transgenic plants [16,31], which is a major advantage for phytosensing. Both transient and stable transgenic expression systems should exhibit congruent expression patterns [16,19,32,33]. Among the four regulatory elements we tested, ERE motif confers the highest expression level while JAR motif confers the lowest (Figures 2, 3 and 4). We therefore, conclude that ERE, PR1 and SARE motifs have more application potential for pathogen phytosensing than the JAR motif.

The use of this expression system for temporary pathogen phytosensing has several benefits when compared to other systems. It avoids the position effects of transgene insertion, requires much less time for measurement of gene expression, and eliminates the possibility of escape of transgenes into the environment. Nevertheless, a few limitations should be considered with regard to our transient expression system for use in pathogen phytosensing. Large variation in expression is a major disadvantage. Leaf size and position, age of plants and growth conditions can affect the transgene expression. The slight discrepancy between inducibility of expression of 4 × JAR RFP and B4 × JARA RFP in response to bacterial pathogens may come from the variation (Figure 3i, j). The OD of infiltration is another major consideration for increasing repeatable results [11,13,24,25,27]. It is always advantageous to adjust the OD of bacterial pathogens to achieve the expected HR (high concentration) or disease symptoms (low concentration) [24-27]. Post-transcriptional gene silencing should also be considered, since plant endogenous defenses can hinder the level and duration of transient expression of reporter genes [34].

## Conclusion

We have tested the suitability, inducibility and potential of gain-of-function analyses of synthetic promoters containing inducible regulatory elements using *Agrobacterium*-mediated transient expression for the purpose of engineering transgenic plants for early detection of pathogen infection. We have demonstrated that agroinfiltration of tobacco leaves has potential to validate the sensitivity and inducibility of the regulatory elements in response to the corresponding phytohormone treatment. Moreover, it is capable of examining the responsiveness of the *pporRFP *reporter to bacterial pathogen infection in both compatible and incompatible interactions. Our results indicate that the *Agrobacterium*-mediated transient expression can be used as a rapid screening tool for *in vivo *analysis of inducible regulatory elements for pathogen phytosensing, allowing high-throughput *in planta *expression screening before conducting stable plant transformation. It could also be utilized for temporary phytosensing which does not require deploying stable transformants.

## Methods

### Plasmid construction

Details of construction of pSK min35SGUS vector consisting of distinct *cis*-acting regulatory elements (REs) without or with CaMV 35S enhancer motifs have been given in our previous study [6]. This vector plasmid was originally constructed for β-glucuronidase (GUS) reporter expression with the ability to swap GUS for fluorescent protein reporters for use in a fluorescent phytosensing system, an *in vivo *system. The pSK min35SGUS vector plasmid containing four copies of distinct REs: pathogenesis-related (PR1), salicylic acid responsive element (SARE), ethylene responsive element (ERE), or jasmonic acid responsive element (JAR) (sequences reported in Ref. 6) were selected for replacing GUS with a red fluorescent protein (RFP) reporter (Figure 1). For enhanced synthetic promoters, version 2 of the CaMV 35S enhancer motif where the RE tetramer was placed between B (-415 to -90) and A1 (-90 to -46) regions of 35S promoter [6] was used in the present study (Figure 1).

The red fluorescent protein *pporRFP *from coral *Porites porites *was used to replace the GUS reporter for potential *in vivo *reporting. Prior to that, PCR-mediated site-directed mutagenesis was performed to remove the *HindIII *restriction site at the position of +138 in *pporRFP *cDNA [GenBank accession number DQ206380] with nucleotide G replaced by A but without changing the encoded amino acid. The GUS reporter of the pSK vector plasmids was then replaced with the RFP reporter (Figure 1). These distinct synthetic promoter-RFP fusion cassettes in pSK vector plasmids were named as: pSK (4 × PR1), pSK (4 × SARE), pSK (4 × ERE), pSK (4 × JAR), pSK (B 4 × PR1 A), pSK (B 4 × SARE A), pSK (B 4 × ERE A), pSK (B 4 × JAR A). Appropriate negative control vectors (empty vectors -4635S RFP and B_A RFP) were produced by digestion of pSK (4 x PR1) and pSK (B 4 × PR1 A)  with XbaI and SpeI (to remove the regulatory element tetramer) followed by self-ligation.

For use in agroinfiltration, each synthetic promoter-RFP fusion cassette was excised from the pSK vector constructs and was inserted into the *SacI*-*HindIII *site of the binary vector pZP222 [35].

### Plants

Tobacco (*Nicotiana tabacum *cv. Xanthi) plants were grown in a growth chamber at 25°C under fluorescent white light in a 16:8 h light/dark cycle. Six-week-old plants were used for agroinfiltration assays.

### Preparation of*Agrobacterium *suspension

*Agrobacterium tumefaciens *strain GV3085 was transformed with each individual construct by electroporation. *A. tumefaciens *containing individual constructs was grown on yeast extract peptone [(YEP) 10 g/L yeast extract, 10 g/L peptone, 5 g/L NaCl, 15 g/L agar] solid medium supplemented with rifampicin (50 mg/L), spectinomycin (200 mg/L), and streptinomycin (50 mg/L) at 28°C for 2 days. One single colony was inoculated in 2 ml YEP liquid medium supplemented with the above-mentioned antibiotics and grown for ~2 h at 28°C. One milliliter of this starter culture was then inoculated in 25 ml YEP liquid medium and grown for overnight at 28°C. *Agrobacterium *cells were collected by centrifugation for 10 min at 3000 *g *and resuspended in 25 ml infiltration medium (50 mg/ml d-glucose, 50 mM MES, 2 mM NaPO_4_.12H_2_O, and 0.1 mM acetosyringone). Centrifugation followed by resuspension in the infiltration medium was repeated for 2 times as above. The bacterial suspension was adjusted to a final OD_600 _of 0.3 for agroinfiltration.

### Agroinfiltration of tobacco leaves

Tobacco plants were removed from the growth chambers and placed under a white fluorescent lamp for 1 h prior to infiltration to open the stomata fully as an aid to infiltration. Infiltration was performed on near fully expanded leaves (~ 5 × 6 cm large, flat, dark green, located in the middle position of the plant) that were still attached to the intact plant. Leaves of the same age on the same branch were used for each experimental test. Each bacterial suspension was infiltrated into leaves of three different plants from the abaxial side of the leaf with a needleless syringe. By infiltration, 100 μl of bacterial suspension was injected into each spot (typically 3-4 cm^2 ^in each infiltrated area). After agroinfiltration, tobacco plants were covered with transparent plastic covers which were sprayed with water and maintained in a growth chamber at 22°C under 16 h light for 24 h. Three biological replicates (i.e., three plants) were used, and the experiments were repeated independently at least three times.

### Biotic and abiotic treatments

For chemical treatments, 48 hrs after the initial agroinfiltration, 4 mM salicylic acid (SA), 4 mg/ml ethephon (an ethylene releasing chemical), or 100 μM methyl jasmonate (MeJA) (all from Sigma, St. Louis, MO, USA) was further infiltrated to the same infiltrated spots. For mock control treatments, leaves were infiltrated with water. For bacterial pathogen treatments, *Pseudomonas syringae *pv. *tomato, P. marginalis*, and *P. syringae *pv. *tabaci*, kindly provided by Dr. Bonnie Ownley, were grown individually at 28°C in tryptic soy broth (TSB) (Becton Dickinson, Sparks, MD, USA) medium overnight. After centrifugation, bacterial cells were resuspended in 10 mM MgCl_2_, followed by centrifugation and were resuspended again. Twenty-four hours after the initial agroinfiltration, each bacterial suspension was further infiltrated at the same infiltrated spots. For mock control treatments, leaves were infiltrated with 10 mM MgCl_2_. The numbers of bacteria was estimated in leaf disks (5 mm in diameter) taken from infiltrated areas at different time points post-infection. The discs were ground in 1 ml of 10 mM MgCl_2_, and serial dilutions were plated out on TSB solid medium. After incubation at 28°C for 24 h, the colonies were counted. All the experiments were repeated independently at least two times.

### Determination of pporRFP expression

Expression of *pporRFP *reporter was measured at time points 0 and 72 h after phytohormone treatments and at time points 0, 24, 48, and 72 h after bacterial pathogen treatments. The treated plants were visualized using an epifluorescence microscope Olympus SZX12 (Olympus, Tokyo, Japan), and the images were captured using imaging software QCapture 2.56 (QImaging, BC, Canada). Fluorescent signal intensity was measured via scanning fluorescence spectrometry using SPEX Fluorolog II spectrophotometer (Horiba Jobin Yvon Inc., NJ, USA). The infiltrated spots were excited at 530 nm, and emission spectra was scanned and recorded from 550 to 640 nm. Intensity was measured at 591 nm in counts per second (cps).

### Data processing and statistical analysis

Background subtraction was applied to each measurement of *pporRFP *expression by using measurements from non-transgenic tobacco as background expression when treated with the biotic and abiotic treatments. Data normalization was then conducted as described [36]. The fold change in the expression of RFP reporter was calculated by using the normalized data at different time points of 24, 48 or 72 h after treatments divided by the normalized data at time point of 0 h. Statistical analyses were conducted by a two-sample *t*-test (*p *< 0.05).

## Authors' contributions

WL designed and constructed all the synthetic promoter constructs, performed a majority of the experiments, analyzed the data, and drafted the manuscript. MM participated in designing the experiments and data analysis, performed the experiments corresponding to the bacterial growth population size, and revised the manuscript. MRR assisted with growing the tobacco plants and preparation of the bacterial inoculums. MHF assisted with data collection corresponding to the fluorescent signal intensity of the *pporRFP *expression. CNS conceived and coordinated the study, assisted with revision to the manuscript, and obtained the funding. All authors read, contributed to, and approved the final version of this manuscript.
